# MitoPhen database: a human phenotype ontology-based approach to identify mitochondrial DNA diseases

**DOI:** 10.1093/nar/gkab726

**Published:** 2021-08-24

**Authors:** Thiloka E Ratnaike, Daniel Greene, Wei Wei, Alba Sanchis-Juan, Katherine R Schon, Jelle van den Ameele, Lucy Raymond, Rita Horvath, Ernest Turro, Patrick F Chinnery

**Affiliations:** Department of Clinical Neurosciences, School of Clinical Medicine, University of Cambridge, Cambridge Biomedical Campus, Cambridge, UK; Medical Research Council Mitochondrial Biology Unit, University of Cambridge, Cambridge Biomedical Campus, Cambridge, UK; Department of Paediatrics, University of Cambridge, Cambridge Biomedical Campus, Cambridge CB2 0QQ, UK; Department of Haematology, University of Cambridge, NHS Blood and Transplant, Cambridge Biomedical Campus, Cambridge CB2 0PT, UK; Medical Research Council Biostatistics Unit, Cambridge Biomedical Campus, Cambridge CB2 0SR, UK; Department of Clinical Neurosciences, School of Clinical Medicine, University of Cambridge, Cambridge Biomedical Campus, Cambridge, UK; Medical Research Council Mitochondrial Biology Unit, University of Cambridge, Cambridge Biomedical Campus, Cambridge, UK; Department of Haematology, University of Cambridge, NHS Blood and Transplant, Cambridge Biomedical Campus, Cambridge CB2 0PT, UK; Department of Clinical Neurosciences, School of Clinical Medicine, University of Cambridge, Cambridge Biomedical Campus, Cambridge, UK; Medical Research Council Mitochondrial Biology Unit, University of Cambridge, Cambridge Biomedical Campus, Cambridge, UK; Department of Medical Genetics, School of Clinical Medicine, University of Cambridge, Cambridge Biomedical Campus, Cambridge, UK; Department of Clinical Neurosciences, School of Clinical Medicine, University of Cambridge, Cambridge Biomedical Campus, Cambridge, UK; Medical Research Council Mitochondrial Biology Unit, University of Cambridge, Cambridge Biomedical Campus, Cambridge, UK; Department of Medical Genetics, School of Clinical Medicine, University of Cambridge, Cambridge Biomedical Campus, Cambridge, UK; Department of Clinical Neurosciences, School of Clinical Medicine, University of Cambridge, Cambridge Biomedical Campus, Cambridge, UK; Medical Research Council Mitochondrial Biology Unit, University of Cambridge, Cambridge Biomedical Campus, Cambridge, UK; Department of Genetics and Genomic Sciences, Icahn School of Medicine at Mount Sinai, New York, NY, USA; Department of Clinical Neurosciences, School of Clinical Medicine, University of Cambridge, Cambridge Biomedical Campus, Cambridge, UK; Medical Research Council Mitochondrial Biology Unit, University of Cambridge, Cambridge Biomedical Campus, Cambridge, UK

## Abstract

Diagnosing mitochondrial disorders remains challenging. This is partly because the clinical phenotypes of patients overlap with those of other sporadic and inherited disorders. Although the widespread availability of genetic testing has increased the rate of diagnosis, the combination of phenotypic and genetic heterogeneity still makes it difficult to reach a timely molecular diagnosis with confidence. An objective, systematic method for describing the phenotypic spectra for each variant provides a potential solution to this problem. We curated the clinical phenotypes of 6688 published individuals with 89 pathogenic mitochondrial DNA (mtDNA) mutations, collating 26 348 human phenotype ontology (HPO) terms to establish the MitoPhen database. This enabled a hypothesis-free definition of mtDNA clinical syndromes, an overview of heteroplasmy-phenotype relationships, the identification of under-recognized phenotypes, and provides a publicly available reference dataset for objective clinical comparison with new patients using the HPO. Studying 77 patients with independently confirmed positive mtDNA diagnoses and 1083 confirmed rare disease cases with a non-mitochondrial nuclear genetic diagnosis, we show that HPO-based phenotype similarity scores can distinguish these two classes of rare disease patients with a false discovery rate <10% at a sensitivity of 80%. Enriching the MitoPhen database with more patients will improve predictions for increasingly rare variants.

## INTRODUCTION

As a group, mitochondrial diseases are amongst the most common inherited disorders, affecting at least 1 in 5000 of the population (1). Recognized for their varied clinical presentation, the symptoms and signs encompass most organ systems, and patients can present at any age from birth to late life. Initially, patients can present with one or two clinical features which overlap with common disorders, including diabetes mellitus, deafness and migraine (2). This presents a major challenge clinically, particularly when different individuals from the same family develop very different problems that do not, at first sight, suggest an inherited disorder.

The investigation of mitochondrial diseases is evolving, with increased availability of genetic testing, particularly exome and whole genome sequencing, leading to faster diagnoses in a greater proportion of patients (3). However, this brings its own challenges, particularly when the implicated genes show considerable nucleotide variation in the general population, as is the case for genes encoded by mitochondrial DNA (mtDNA). Distinguishing new pathogenic mutations from rare polymorphisms can be difficult based on sequence data alone. The independent occurrence of the same mutation in patients with a similar phenotype provides the strongest evidence of pathogenicity (4), but this is particularly challenging for mitochondrial diseases, where the range of possible phenotypes is so broad and overlaps with common disorders. For example, when is migraine or diabetes part of the phenotype or just a coincidental finding?

The past 30 years have seen a massive expansion of mitochondrial diagnostics, underpinning the discovery of new pathogenic mutations and new phenotypes, proceeding on a case-by-case basis for individual families or small patient cohorts (5). This new knowledge presents an opportunity for a more systematic approach to characterizing existing mitochondrial disorders and identifying new such disorders, which has previously been achieved successfully in the context of other rare diseases (6). This is critically dependent on systematic phenotyping using a controlled vocabulary such as the Human Phenotype Ontology (HPO) (7).

Based on current understanding, mtDNA mutations account for two-thirds of mitochondrial disease diagnoses, where the vast majority are due to single nucleotide variants (SNVs) of mtDNA (mtSNVs) (1). Here, we present the MitoPhen database of published mtDNA disease patients with mtSNVs or small insertions/deletion variants (indels). We assigned an average of 7.1 HPO terms to the 3682 affected individuals in the database with HPO terms (14 affected individuals could not be assigned HPO terms due to a lack of information in the relevant publications), each harboring one of 89 established pathogenic mtDNA variants, from a review of 676 publications. The HPO terms covered all major organ systems, enabling a comprehensive description of the spectrum of clinical phenotypes associated with each variant. Unlike previous analyses using marginal frequencies of phenotypic terms (8), we analyzed the within-individual co-occurrence of HPO terms. We also recorded the heteroplasmy levels in muscle, blood and other biological materials where available, which allowed us to identify associations between heteroplasmy levels of specific variants and the odds of particular HPO terms being present. The database also enabled the identification of under-recognized but recurrent clinical features in patients carrying particular mtDNA variants. Finally, we computed measures of phenotypic similarity between variant carriers in MitoPhen and 1160 independently coded rare disease patients, highlighting the potential utility of MitoPhen in a clinical genetic setting.

## MATERIALS AND METHODS

### Identification of pathogenic mtDNA variants

We initially considered 111 mtDNA variants categorized as pathogenic in MITOMAP (86 variants, February 2019) (9) or ClinVar (57 variants) (10). A systematic review of the literature was carried out for each variant using MITOMAP, PubMed and Google Search (Supplementary Methods), enabling an independent re-classification of pathogenicity based on established criteria developed specifically for the evaluation of mtDNA mutations (11). These criteria resemble those published by the American College of Medical Genetics (12) as indicated, with the addition of functional criteria specific for mitochondrial diseases. The ACMG guidelines (12) were designed primarily for nuclear variants. Several of the criteria cannot easily be applied to mtDNA variants—for example the *de novo* status may be less clear cut because a pathogenic variant may be inherited from a very mildly affected mother with a low level of heteroplasmy. Most mtDNA variants in protein coding genes are missense rather than loss of function so evidence criteria for truncating variants will be less useful for mtDNA diseases (12). In brief, ‘pathogenic mtDNA variants’ required: (i) two or more independent reports in patients with suspected mitochondrial disease (ACMG PS4); (ii) biochemical or histochemical evidence of mitochondrial dysfunction including fibroblast or other cell lines (ACMG PS3); (iii) functional evidencing including documentation of single fiber studies, cybrid [cytoplasmic hybrid] or other functional study models such as *Escherichia coli* or *Sacchromyces*, or the use of computational models to demonstrate a protein structural implication of the variant, or steady state level experiments (ACMG PS3); (iv) segregation between patient tissues and/or within the family (ACMG PP1) and (v) evolutionary conservation, using the phastCons program (ACMG PP3) (13). Four mtSNVs were only reported in one family but were included due to functional evidence of pathogenicity. We did not factor in the evolutionary conservation of amino acid residues given previous evidence that it did not add further weight to determining pathogenicity in mitochondrial disease (14,15). We also did not consider cybrid data where multiple mtSNVs within the same cell line made it difficult to establish the functional effect of each variant (eg. m.4160T > C and m.11253T > C (16,17)). We did not study mtDNA large-scale deletions.

### Curation of the patient data

Next, we performed a systematic review of the literature reporting each pathogenic mtDNA variant until August 2019 including MITOMAP listings, PubMed and Google searches using relevant text strings (e.g. ‘8344’ + ‘mitochondrial’ + ‘clinical’), as previously described (18). Articles that did not include patient-specific information such as review articles were excluded. Individual level data from each article was added to a relational database incorporating the mitochondrial variant and the phenotype data, coded using non-redundant HPO terms (HPO web interface, March 2020 release) (19). Given that the HPO is an ontology of phenotype abnormalities and not diseases, it does not list syndrome names. Therefore, where a syndrome was listed in a publication, the closest related HPO term was consistently chosen (20). For example, Leigh syndrome was coded as ‘necrotizing encephalopathy’ as the closest relevant term because this is a hallmark of Leigh syndrome as listed in OMIM (20). If a patient was labelled as having Mitochondrial Encephalopathy, Lactic Acidosis and Stroke-like episodes (MELAS), then these specific HPO terms were recorded, unless further phenotype information was available in the publication. Additional data gathered included: sex, age at onset, clinical features at onset, percentage of cytochrome *c* oxidase (COX) deficient muscle fibers, percentage of ragged-red muscle fibers, heteroplasmy level and tissue sampled (up to three tissues), and generation of the individual relative to the proband in the maternal lineage (Supplementary Methods). All the individuals were given a unique identifier, and the proband identifier was used to group family members within the dataset.

### Phenotypic spectrum associated with mtDNA variants

Using OntologyX (21), we identified all the ancestors of the HPO terms assigned to each individual, enabling a high level analysis of the affected organ systems, such as ‘Abnormality of the nervous system’. To compute a phenotypic similarity score between two sets of individuals in MitoPhen (e.g. those corresponding to two sets of probands carrying different mtDNA variants), we first considered the similarity between two HPO terms. We used Lin's expression for the similarity between terms (22), which depends on the ‘information content’ (IC) (i.e. negative log frequency) of terms in a particular context (here we used the set of probands in MitoPhen) and which ranges between zero and one. This expression is given by $Lin\ ( {t1,\ t2} ) = \ \frac{{2{\rm{\ IC}}( {{\rm{MICA}}( {t1,\ t2} )} )}}{{{\rm{IC\ }}( {t1} ) + {\rm{IC}}( {t2} )}}$ where *t1* and *t2* are the two terms being compared, *IC(t)* is the information content of term *t* and *MICA(t1, t2)* is the most informative common ancestor of terms *t1* and *t2*. The asymmetric similarity between two sets of HPO terms S1 and S2 (e.g. those corresponding to two individuals) was obtained by computing the best match in S2 for each term in the S1 and taking the mean. The symmetric similarity between the two sets was computed by taking the mean of the asymmetric similarities computed in each order (23). Finally, the phenotypic similarity score between two sets of individuals was computed as the mean symmetric phenotypic similarity across all pairs of individuals made up of one individual from each set.

### Association between heteroplasmy levels and HPO terms

For each variant and HPO term, we performed logistic regression of a binary response indicating presence or absence of the HPO term on blood and muscle heteroplasmy levels for the variant in probands. The overall expected false discovery rate at *P*-value threshold *t* was computed by dividing the expected number of *P*-values less than *t*, as obtained by permutation of the heteroplasmy levels (within variant and tissue), by the observed number of tests yielding a *P*-value less than *t*.

### Identifying under-recognized phenotype abnormalities associated with mtDNA variants

To identify HPO terms that were over-represented in affected carriers of particular variants, we applied the following procedure. For each variant, we compared the frequency of each term in probands carrying the variant with the frequency of the term in probands not carrying the variant. We made these comparisons in MitoPhen as a whole and also within each publication. If one-sided Fisher's exact *P*-values were <10^–6^ across all publications and in at least two independent publications, then we declared that term to be significantly enriched in carriers of that variant. Requiring significance in two publications guarded against author-specific biases in the reporting of phenotypes.

The HPO terms that were enriched in carriers of particular variants were further deemed to be *under-recognized* if they were absent from the Orphanet terms associated with these variants (24). To achieve this, we first had to identify the clinical syndromes in OMIM associated with each of the 89 pathogenic mtDNA variants (this is not possible using Orphanet directly because Orphanet only contains gene-level, not variant-level, phenotype data). We then mapped the variant-specific OMIM syndromes to Orphanet diseases and retrieved the corresponding HPO terms. This gave a list of HPO terms associated with each variant in the literature. For instance, *m.3243A > G* is associated with a number of syndromes in OMIM such as ‘MELAS’, and these syndromes were cross-referenced in Orphanet in order to identify the HPO terms associated with this variant (24).

### Phenotype similarity between members of independently coded patient cohorts and MitoPhen

We assessed whether phenotypic similarity to individuals in the MitoPhen database could be used to distinguish mtDNA disorders from rare diseases due to non-mitochondrial nuclear genetic disorders. We compiled a test dataset comprising 77 patients with a confirmed mtDNA disorder and 1,083 individuals with a confirmed non-mitochondrial nuclear genetic rare disease from the NIHR BioResource Rare Diseases study (6) (Supplementary Table S1). HPO terms for the NIHR BioResource participants were previously applied by clinicians without a template. To compute the similarity between a test patient and MitoPhen, we considered the similarity between the patient and each of the variants in MitoPhen. To allow for within-variant phenotypic heterogeneity, for each variant, we took the mean similarity between the test patient and the five most similar MitoPhen probands with the variant (using the asymmetric similarity measure defined above). The 48 MitoPhen variants with fewer than five probands were excluded. We then assigned the maximum of the mean similarity score over variants to the test patient. A high score meant that a reasonably sized subset (we arbitrarily chose five) of probands carrying the same variant existed in the MitoPhen database who were very similar to the test patient.

## RESULTS

### Pathogenic variants and individuals within the MitoPhen database

**Figure 1.**
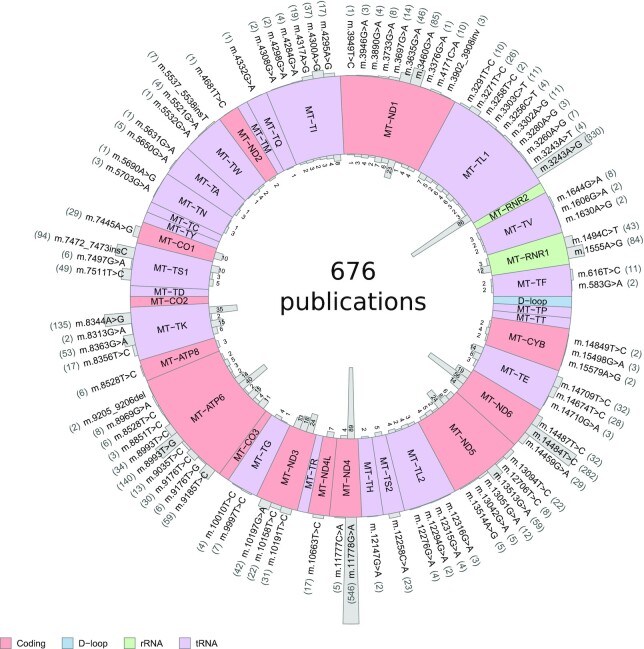
Confirmed pathogenic variants in the mitochondrial genome. Eighty-nine pathogenic mtDNA variants and the corresponding mtDNA gene positions. The number of published affected individuals for each variant is represented by outward-facing grey bars, with the number shown in brackets, and the number of publications reporting these individuals are represented as inward-facing grey bars. The mtDNA genes are colored according to coding category: pink represents coding genes, purple represents tRNA genes, green represents rRNA genes and the D-loop is shown in blue.

Of the 111 mtDNA variants we considered, 89 fulfilled our criteria for pathogenicity (4 indels, 85 SNVs), spanning 27 genes (Supplementary Table S2). Forty variants were in mtDNA coding regions, two in *MT-*RNR1, and 47 in 15 of the 22 mtDNA-encoded tRNAs genes (Figure 1). 1352 publications were found in total reporting the 89 variants. Only 676 of the publications were used to populate the MitoPhen database after exclusion of papers which did not report individual-level data. This includes data from 6688 individuals in 1424 families. Forty individuals (0.6%) harbored two confirmed pathogenic mtSNVs. Of the 6688 individuals, 2955 (44%) were male and 3624 (54%) were female (the sex was not available for 2%); 3696 (55%) were recorded as being clinically affected and 2956 (44%) as unaffected (the clinical status was unclear for 1%). Data on multiple family members were available for 818 (57%) of the 3379 probands. The number of affected individuals harboring each variant is shown in Figure 1. Heteroplasmy levels in at least one tissue were available for 2209 (60%) of affected individuals (Supplementary Table S3).

### Phenotypic spectrum of individuals in the MitoPhen database

**Figure 2.**
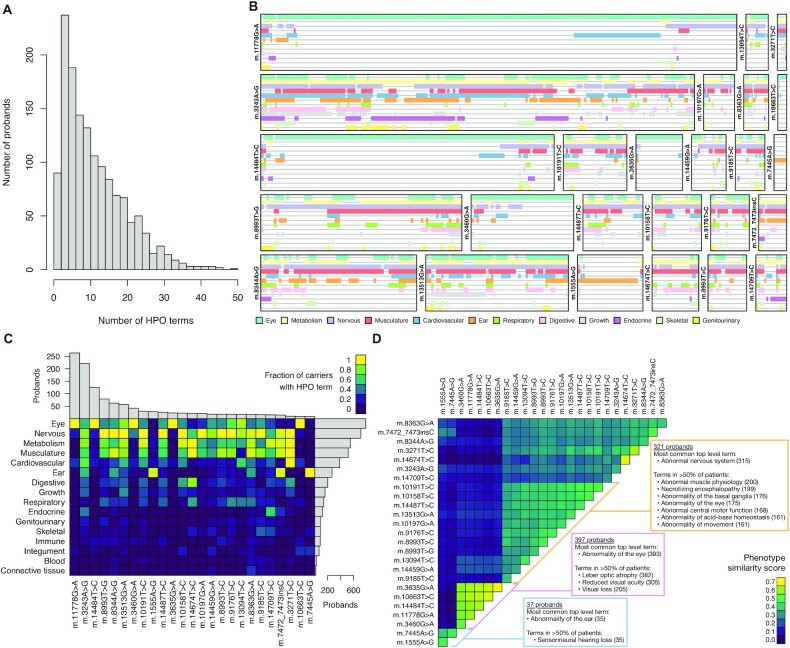
Human Phenotype Ontology terms curated from 6689 individuals harboring 89 pathogenic mtDNA variants. (**A**) Histogram of the number of non-redundant HPO terms assigned to the probands. (**B**) Phenotypes of the probands affected by the 25 mtDNA variants carried by the greatest number of probands. Each box represents a different variant. Within each box, individuals are represented by vertical arrangements of colors corresponding to top level HPO terms that they have been assigned. (**C**) Heatmap of the fraction of probands carrying each of the 25 mtDNA variants who have abnormalities in each of the top level HPO terms. The histograms show the number of probands carrying each variant and having each phenotypic abnormality. (**D**) Heatmap of the phenotype similarity score comparing sets of probands carrying each of the 25 mtDNA variants. The rows and columns have been arranged by hierarchical clustering. For each of the three apparent clusters, the most common HPO term and the most specific terms in at least 50% of the probands in the cluster are listed.

We recorded 26 348 HPO terms across 3800 individuals, which includes 118 individuals noted as unaffected or asymptomatic, consisting of 1747 different terms. The entire dataset is available on-line through www.mitophen.org. The mean number of terms per proband was 11.4 (Figure 2A). Figure 2B demonstrates the heterogeneous nature of mitochondrial disease, with nervous system, musculature, metabolism, cardiovascular, ear and eye terms being noted in different combinations in the probands carrying the 25 most commonly reported mtDNA variants. Clustering of system involvement is also apparent, which is only possible through amalgamation of published records, but does not work at the individual level. For example, *m.11778G > A* shows a distinct group of patients with both eye and heart involvement, and there are clusters of patients with multisystem involvement including the digestive system within *m.3243A > G*. This is further appreciated in Figure 2C which groups the data by system and variant in descending order of frequency. The separate 1498 HPO terms (grouped in 16 systems) are listed as proportions of affected individuals for each of the 25 common mtDNA variants, showing the differences between how common certain features of mitochondrial disease are, such as ‘hearing impairment’ (HP:0000365) which occurs in less than 10% of affected cases in 12 of these variants (Supplementary Table S4).

Hierarchical clustering of the HPO-coded phenotypes using phenotype similarity scores identified three main groups amongst the 25 most common variants (Figure 2D), corresponding to: (i) mtDNA variants causing isolated deafness (*m.1555A > G, m.7445A > G*); (ii) mtDNA variants causing optic neuropathy (*m.3635G > A, m.10663T > C, m.14484T > C, m.11778G > A, m.3460G > A*) and (iii) mtDNA variants causing a broad range of abnormalities of the nervous system (m.10191T > C, m.10158T > C, m.14487T > C, m.13513G > A, m.10197G > A, m.9176T > C, m.8993T > C, m.8993T > G, m.13094T > C, m.14459G > A, m.9185T > C).

### Association between heteroplasmy level and phenotype

**Figure 3.**
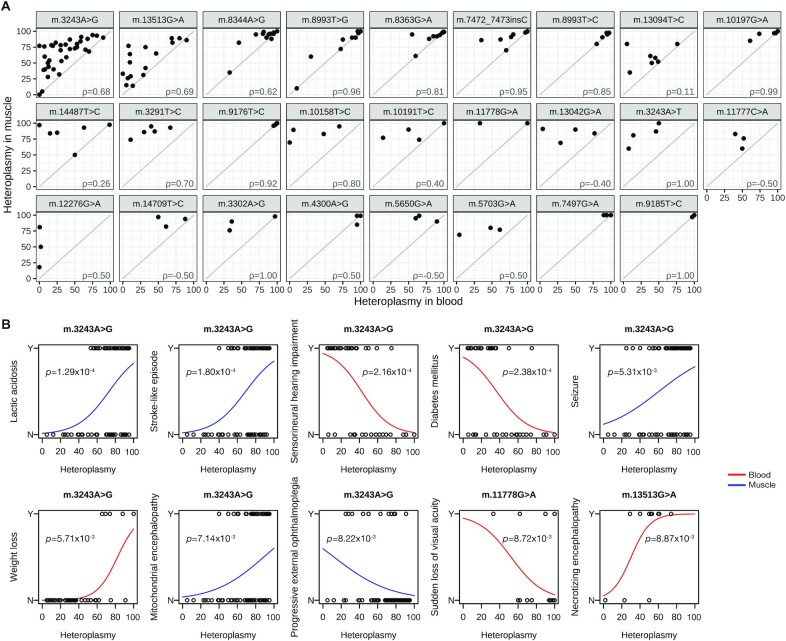
Heteroplasmy levels and associated phenotypes. (**A**) Scattergrams of heteroplasmy levels in muscle and blood. Variants are shown when there are at least three probands with data available. The Spearman correlation coefficient ρ is shown in each scattergram. Only eight out of 179 individuals had a lower heteroplasmy level in muscle than in blood. (**B**) Fitted logistic regression curves showing correlations between specific HPO terms and variant heteroplasmy levels (%) in muscle (red) or blood (blue) (in each case, *P* < 0.01). By permutation, we expected 1.86 *P-*values less than 0.01 under the null, yielding an expected false discovery rate of 15.5%. Note that two significant associations with more general HPO terms than those shown in the panel have been omitted (between m.3243A > G in muscle and both ‘Ophthalmoplegia’ and ‘External ophthalmoplegia’).

Heteroplasmy levels were only loosely correlated with each other, with lower levels in peripheral blood and variant-to-variant differences in the relationship between blood and muscle (Figure 3A). The logistic regression analyses between individual HPO terms and heteroplasmy levels revealed positive relationships with terms associated with MELAS such as stroke-like episode, whereas there were negative relationships in blood heteroplasmy level with sensorineural hearing impairment and diabetes mellitus in *m.3243A > G* (Figure 3B). There was also a positive relationship between blood heteroplasmy and the presence of Leigh syndrome (defined as ‘necrotizing encephalopathy’) in *m.13513G > A* (Figure 3B).

### Under-recognized phenotypes in commonly reported mtSNVs

**Figure 4.**
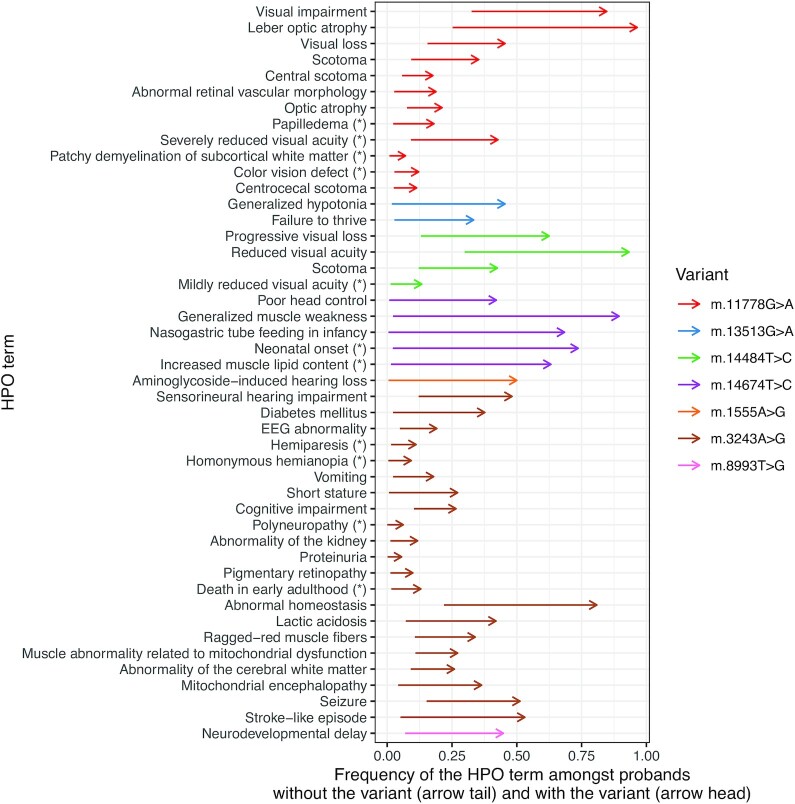
Variant-specific enrichment of HPO terms. HPO terms exhibiting strong evidence of enrichment in probands carrying particular variants are shown. Each arrow leads from the frequency (as a proportion of 1) of the term amongst all probands in the MitoPhen database who do not carry the corresponding variant (arrow tail) to the frequency amongst those carrying the variant (arrow head). For example, for m.11778A>G, nearly 100% of individuals in the MitoPhen database carrying this variant have 'Leber Optic Atrophy' listed as a phenotype, and 25% of individuals in MitoPhen who do not harbour m.11778A>G also have 'Leber Optic Atrophy' listed, reflecting the other mtDNA variants known to cause this disorder that are included in the database. Asterisks are used to denote under-recognized terms, specifically those that are not listed by Orphanet as associated with the syndrome caused by the variant.

We identified 46 enriched HPO associations with seven frequently reported variants: *m.11778G > A, m.3243A > G, m.14484T > C, m.8993T > G, m.13513G > A, m.14674T > C, m.1555A > G*; 11 of these were not listed in Orphanet with the associated syndromes (Figure 4). These HPO terms can, however, be explained clinically, for example ‘color vision defect’ in *m.11778G > A*, and ‘polyneuropathy’ in *m.3243A > G* are known. The 46 HPO terms do highlight how specific certain phenotypes are for certain variants—for instance, ‘nasogastric tube feeding in infancy’ is recorded frequently in patients with the *m.14674T > C* mutation and reversible infantile mitochondrial myopathy, however this term is found rarely within the rest of MitoPhen (Figure 4).

### HPO-based classification of mitochondrial disease patients

**Figure 5.**
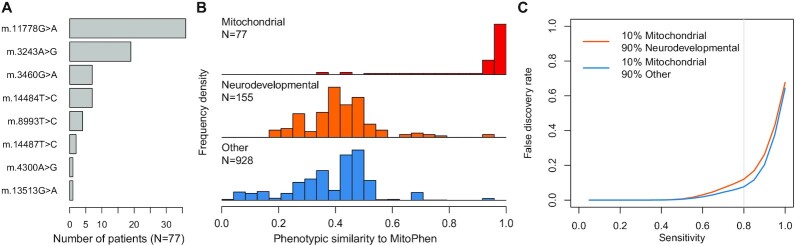
Predicting the cause of rare disease in patients using phenotypic similarity to MitoPhen. (**A**) Distribution of causal variants in the independent mtDNA disease cohort of 77 individuals. (**B**) Distribution of phenotypic similarity scores of each cohort, where the non-mitochondrial disease cohort was partitioned into neurodevelopmental diseases and other diseases caused by nuclear genetic mutations. (**C**) False discovery rate for predicting mtDNA disease, thresholding phenotypic similarity to achieve a given sensitivity. FDR was estimated separately for the neurodevelopmental and non-neurodevelopmental sections of the non-mitochondrial disease cohort with known nuclear genetic diseases. Bootstrap sampling was used to achieve a mixture of 10% mitochondrial DNA disease for each estimated FDR. The vertical grey line indicates a sensitivity of 0.8.

Next, we compared the mean phenotype similarity scores between the 77 patients with confirmed mtDNA disease (Figure 5A) and MitoPhen with the corresponding scores for 155 cases with a neurodevelopmental disorder and 928 cases with various other rare diseases due to nuclear gene mutations (Figure 5B). Although there was overlap, the mtDNA disease patients tended to have higher phenotypic similarity scores. Using mixed samples of 200 test cases (10% mtDNA disease patients, 90% neurodevelopmental or other, chosen at random from the two groups), we achieved mean false discovery rates of 0.12 and 0.08 for neurodevelopmental and other rare disease cases respectively, with a sensitivity of 80% for identifying the mtDNA disease patients using phenotype similarity scores computed through MitoPhen (Figure 5C).

## DISCUSSION

Assembling and curating a database of 26 348 HPO terms in 3800 individuals with mitochondrial disease caused by 89 different mtSNVs provides an open reference dataset facilitating the diagnosis of mtDNA diseases. The online patient-centric database www.MitoPhen.org allows clinicians to identify previously described individuals with the same or a similar cluster of HPO terms, supporting a clinical diagnosis of mtDNA disease. In addition, the link to underlying mtDNA mutations allows molecular diagnostic labs to validate genotype–phenotype associations. MitoPhen contains >10% of all of the terms contained within the HPO (7). This emphasizes the breadth and complexity of phenotypes associated with mitochondrial disease. It would be extremely challenging for any one clinician to commit all of this information to memory. This adds weight to the importance of developing objective methods that harnesses the growing body of clinical data when trying to diagnose all but the most typical mitochondrial disorders.

Although there are always concerns that a literature-based dataset is influenced by ascertainment or reporting bias, and the subjectivity of individual clinicians, it is reassuring that the proportion of patients with common mtDNA variants included in our dataset corresponds closely with epidemiological data (1), indicating that the MitoPhen database is likely to reflect mtDNA diseases in the population. To gauge the robustness of our method of capturing HPO phenotype data from the literature, we selected 10 random publications from the MitoPhen database and had an independent reviewer record HPO phenotypes for the same set of 79 patients. 84% of the HPO terms identified by the first reviewer were recorded by the second reviewer, and 86% of the HPO terms identified by the second reviewer were recorded by the first reviewer, indicating a strong concordance between individual classifiers. Further reassurance comes from the hierarchical clustering of phenotypes, which independently identified mutations associated with multiple or single disease phenotypes (Figure 2B and C). In addition, when looking at phenotypic traits associated with specific variants, we only included phenotypes seen in multiple publications to guard against author-specific bias (Figure 4).

The MitoPhen data confirms findings that maternally inherited diabetes, deafness, and external ophthalmoplegia are more common in patients with lower blood heteroplasmy levels, while seizures and encephalopathy are more common in patients with higher blood and muscle heteroplasmies (Figure 3B) (25). This has been observed before (26), and could reflect a co-segregating nuclear genetic modifier in specific families influencing the distribution of mtDNA heteroplasmy at a cellular level. Finally, the entire dataset of HPO terms (Supplementary Table S4) enables clinicians to look up the frequency of under-recognized phenotypes. This can be used in various ways, including determining whether a new feature emerging during the disease course is likely to be part of the underlying diagnosis, or whether it is likely to be unrelated to the mitochondrial disease.

Using Lin's expression for the similarity between HPO terms, we show that phenotype similarity scores can be used to distinguish patients with specific mtDNA mutations from rare disease controls. This was only possible for the most common mutations, such as m.3243A > G found in 468 affected individuals. Thus, even large national referral centers will find it challenging to use this approach in isolation, placing greater emphasis on the importance of sharing both molecular *and* clinical data to improve diagnostic pipelines. Based on the test-cases studied here, pooling data from across the globe will enable a similar approach for rarer mtDNA mutations and improve the sensitivity and specificity for a given threshold similarity score. Enriching the dataset with more HPO terms associated with specific variants will likely improve the specificity of any predictions (27), which may be specific to different levels of heteroplasmy, and may change during the disease course for an individual patient. However, the real value of this approach will be to use phenotype similarity scores to identify new mtDNA disease mutations based on a phenotypic match. For example, by using MitoPhen, it will be possible to objectively identify individuals within an HPO database who have an ‘m.3243A > G-like’ phenotype in an unbiased way, and to compare their nuclear and mtDNA genotypes. Building a larger database would allow similar lines of enquiry across a range of mtDNA disease, increasing the potential for novel molecular diagnoses.

Although we used similarity scores based on Lin's expression, other scoring approaches could also be used (28). Making the entire dataset available allows other methods to be tested in a similar way, and will hopefully catalyze the development of new, more powerful algorithms. It is also worth noting that we limited our analysis to 89 mtDNA variants where there was convincing evidence of pathogenicity. A larger number of mtDNA variants have been associated with disease, and our approach could be used to validate their pathogenicity, particularly when functional data is lacking. We did not set out to study mtDNA deletions, in part because the associated clinical phenotypes are well defined and more limited than missense variants. We also did not curate data from patients with nuclear mitochondrial gene defects, largely because the number of published cases is substantially less than for mtDNA mutations. However, expanding the dataset to include both of these groups will enhance the potential utility, and allow an objective comparison between different molecular causes of mitochondrial disease. A more comprehensive reference dataset could be used to identify phenotypic matches for nuclear-encoded mitochondrial disorders, and potentially advance our understanding of interacting loci including phenotypes influenced by the nuclear or mitochondrial genetic background. This will be important, because of emerging evidence that some mtDNA variants cause disease in some populations, but not others (such as *m.4295A > G* (10)).

The entire dataset is available for download through www.mitophen.org. The website includes search features to enable clinicians to group patients by mtDNA variant or HPO terms. We aim to update MitoPhen when new literature emerges and we are undertaking a similar approach for nuclear-encoded mitochondrial diseases.

## DATA AVAILABILITY

The data and code relating to this manuscript are available through the web-links below.

## WEB RESOURCES

MitoPhen database https://www.mitophen.org.

## Supplementary Material

gkab726_Supplemental_Files
