# Supplementary material for: MitoPhen database: a human phenotype ontology-based approach to identify mitochondrial DNA diseases
Source: Nucleic Acids Res. 2021 Aug 24;49(17):9686–95. doi: 10.1093/nar/gkab726 (PMC8464050; doi:10.1093/nar/gkab726)
Supplement: gkab726_Supplemental_Files [file gkab726_supplemental_files.zip › Supplementary Information.docx]

# Supplementary Information

**Supplementary Methods. Definition of pathogenic mtDNA variants and curation of patient phenotypes.**

**Table S1: Patients in the NIHR BioResource - Rare disease study with a non-mitochondrial nuclear genetic disorder included in this study.**

**Table S2: 89 mtDNA variants and published evidence of pathogenicity.** Criteria for pathogenicity [9]: conservation status high (Y) or not (N) using phastCons [10]; histochemical changes in keeping with mitochondrial DNA (mtDNA) defect; biochemical changes in keeping with mtDNA change; single fibre study available and supportive of pathogenicity (Y) or not (N); fibroblast study showing respiratory chain defect, cybrid cell culture or other study of functional impact such as steady-state or structural assessment of protein, evidence of segregation of variant within maternally related family members or between tissues. Data included from MITOMAP [7]: homoplasmy, heteroplasmy (‘+’ indicates evidence available, ‘-‘ means unavailable), MITOMAP status of confirmed pathogenicity (Cfrm) or reported, frequency data derived from GenBank sequences, and total references listed in MITOMAP. Pubmed identifiers (PMID) are listed for publications detailing studies assessing functional impact of variant, and additional supporting studies are listed in the final column. Comments are available where functional impact is shown through studies other than single fibre analyses or cybrid cell lines.

**Table S3: Summary of MitoPhen database demographics.** Listed characteristics are: Gender (male, female or not done – ND), Disease status (affected, unaffected, or not done- ND), Generation relative to proband (where proband is generation ‘0’, mother is ‘-1’, offspring are ‘1’ and so on). Numbers of affected patients with heteroplasmy and human phenotype ontology (HPO) data are listed, as well as the number of probands with one or more maternal relatives in the database.

**Table S4: 25 most frequent variants within MitoPhen and HPO terms listed in association with proportions in cases noted*.*** Data shows the proportion affected, not the percentage. All 1498 HPO terms found in the 25 most frequent mtDNA variants within MitoPhen are listed in the first column, in numerical order. The 25 mtDNA variants are shown in the following columns with proportion of all affected cases with each variant, where the HPO term is found. For a number of variants, the proportion is 0 because the HPO term is not found in affected cases with that variant. To illustrate, ‘multicystic kidney dysplasia’ (HP:0000003) is only seen in one case diagnosed with m.3243A>G, whereas ‘external ophthalmoplegia’ (HP:0000544) is seen in 15 variants in varying proportions of affected patients.
