# Supplementary material for: MitoPhen database: a human phenotype ontology-based approach to identify mitochondrial DNA diseases
Source: Nucleic Acids Res. 2021 Aug 24;49(17):9686–95. doi: 10.1093/nar/gkab726 (PMC8464050; doi:10.1093/nar/gkab726)
Supplement: gkab726_Supplemental_Files [file gkab726_supplemental_files.zip › Supplementary_Methods_R1_FINAL-.docx]

# Supplementary Methods

## Definition of pathogenic mtDNA variants

We reviewed each variant, using online published literature through PubMed and Google searches, documenting ‘yes’ as ‘Y’ or ‘no’ as ‘N’ based on previously published criteria (1):

1) Two or more independent laboratories report the variant,

2) Biochemical or histochemical evidence of mitochondrial dysfunction (including the use of fibroblast or other blastoid cell lines),

3) Functional study evidencing effect of mtDNA variant*: includes documentation of single fibre studies, cybrid or other functional study models such as E. coli or Sacchromyces, or the use of computational models to demonstrate a protein structural implication of the variant, or steady state level experiments,

4) Segregation between patient tissues and/or within the family,

5) Evolutionary conservation, using the phastCons program (2).

*This was the determining factor for whether there was enough evidence for the variant to be listed as definitely pathogenic or not.

Although evolutionary conservation was documented, it did not factor in determining pathogenicity as previously noted in well-established yet poorly-conserved affected bases such as in the case of m.8344A>G (3,4).

In the situation where a variant has only been published in one patient, if there was robust functional evidence to support pathogenicity of the variant (cybrid or single fibre studies), then the variant was listed within the pathogenic group. *In vivo* or *in silico* studies were accepted as a form of evidence for functional impact of the variant, as long as there was other evidence of biochemical or histochemical defects.

Variants which lacked any form of published functional study of impact were excluded from the pathogenic list, including those where a cybrid study was published but there were multiple variants within the same cell line making it difficult to establish the functional effect of each variant, as was the case with m.4160T>C and m.11253T>C (5,6).The fully annotated tables of variants and PubMed identifiers for selected studies evidencing functional impact is available in the supplementary tables.

## Curation of patient phenotypes

We used a previously described methodology (7) of online searches for published information per variant. This approach meant that methodology, mechanistic, review articles and articles discussing cohort data were excluded if patient-specific information was unavailable. If the full text was not available, then the abstract was used as long as individual patient data could be extracted. Tables and figures within each publication were also used as long as it was possible to identify the individual’s phenotypic data from these. Phenotypic data was recorded in the chronological order per case history where possible, so this meant that symptoms at onset could be highlighted. Specific ages of onset are usually given in publications; however, we have interpreted these broadly into ‘neonatal onset’ (if symptoms before 28 days of life), ‘infantile onset’ (one month to one year old), ‘childhood onset’ (one year old to 12 years old), ‘juvenile onset’ (13 years old to 18 years old), or ‘adult onset’ (over 18 years old) for the purposes of the Human Phenotype Ontology (HPO, March 2020 release) (8). The term ‘death in early adulthood’ was noted in any cases where death had occurred before the age of 60 years.

**References**

1. Yarham, J.W., Al-Dosary, M., Blakely, E.L., Alston, C.L., Taylor, R.W., Elson, J.L. and McFarland, R. (2011) A comparative analysis approach to determining the pathogenicity of mitochondrial tRNA mutations. *Hum Mutat*, **32**, 1319-1325.

2. Siepel, A., Bejerano, G., Pedersen, J.S., Hinrichs, A.S., Hou, M., Rosenbloom, K., Clawson, H., Spieth, J., Hillier, L.W., Richards, S. *et al.* (2005) Evolutionarily conserved elements in vertebrate, insect, worm, and yeast genomes. *Genome Res*, **15**, 1034-1050.

3. Shoffner, J.M., Lott, M.T., Lezza, A.M., Seibel, P., Ballinger, S.W. and Wallace, D.C. (1990) Myoclonic epilepsy and ragged-red fiber disease (MERRF) is associated with a mitochondrial DNA tRNA(Lys) mutation. *Cell*, **61**, 931-937.

4. Yarham, J.W., Blakely, E.L., Alston, C.L., Roberts, M.E., Ealing, J., Pal, P., Turnbull, D.M., McFarland, R. and Taylor, R.W. (2013) The m.3291T>C mt-tRNA(Leu(UUR)) mutation is definitely pathogenic and causes multisystem mitochondrial disease. *Journal of the neurological sciences*, **325**, 165-169.

5. Wong, R.C.B., Lim, S.Y., Hung, S.S.C., Jackson, S., Khan, S., Van Bergen, N.J., De Smit, E., Liang, H.H., Kearns, L.S., Clarke, L. *et al.* (2017) Mitochondrial replacement in an iPSC model of Leber's hereditary optic neuropathy. *Aging (Albany NY)*, **9**, 1341-1350.

6. Cruz-Bermudez, A., Vicente-Blanco, R.J., Hernandez-Sierra, R., Montero, M., Alvarez, J., Gonzalez Manrique, M., Blazquez, A., Martin, M.A., Ayuso, C., Garesse, R. *et al.* (2016) Functional Characterization of Three Concomitant MtDNA LHON Mutations Shows No Synergistic Effect on Mitochondrial Activity. *PLoS One*, **11**, e0146816.

7. Bandelt, H.J., Salas, A., Taylor, R.W. and Yao, Y.G. (2009) Exaggerated status of "novel" and "pathogenic" mtDNA sequence variants due to inadequate database searches. *Hum Mutat*, **30**, 191-196.

8. Köhler, S., Gargano, M., Matentzoglu, N., Carmody, L.C., Lewis-Smith, D., Vasilevsky, N.A., Danis, D., Balagura, G., Baynam, G., Brower, A.M. *et al.* (2021) The Human Phenotype Ontology in 2021. *Nucleic Acids Res*, **49**, D1207-d1217.
